# Falls in Huntington's Disease: A Cross‐Sectional Analysis of Clinical Features and Potential Contributors

**DOI:** 10.1002/mdc3.70754

**Published:** 2026-07-30

**Authors:** Arthur Pena Ferreira, Sarah Edith Souza de Assis Leão, Ricardo Oliveira Horta Maciel, Débora Palma Maia, Aline Alvim Scianni, Francisco Eduardo Costa Cardoso

**Affiliations:** ^1^ Movement Disorders Unit, Neurology Service, Department of Internal Medicine The Federal University of Minas Gerais Belo Horizonte Brazil; ^2^ Division of Neurology, Department of Medicine Sunnybrook Health Sciences Centre, University of Toronto Toronto Ontario Canada; ^3^ Program in Rehabilitation Sciences, Department of Physical Therapy Universidade Federal De Minas Gerais Belo Horizonte Brazil

**Keywords:** chorea, falls, Huntington's disease, neuroleptics, postural instability

## Abstract

**Background:**

Falls occur across all stages of Huntington's disease (HD) and are associated with poor quality of life and injury. However, there is limited information on falls in HD.

**Objective:**

The aim was to investigate the clinical features potentially associated with falls in HD.

**Methods:**

We conducted a cross‐sectional, analytical observational study, including consecutive patients with genetically confirmed symptomatic HD, and assessed clinical features, fall characteristics, fear of falling, movement disorder phenomenology, gait characteristics, balance, and cognitive and neuropsychiatric symptoms. Those who had experienced ≥2 falls in the past 6 months were considered fallers. Stepwise forward logistic regression was performed to determine the variables related to recurrent falls.

**Results:**

We included 40 individuals, of whom 24 (60%) were considered recurrent fallers. The nonfallers (75%) and fallers (79%) had high fear of falling rates. Seventy‐two percent of falls occurred indoors, and 76% were classified as intrinsic. The dose of neuroleptics was higher in the fallers group (10.0 vs. 5.85, *P* = 0.028). This group also exhibited a higher prevalence of balance disorders, chorea, and executive cognitive impairment than the nonfallers group. No significant differences were observed in the spatiotemporal gait parameters studied. The regression analysis revealed that only the Berg Balance Scale scores were retained in the model (odds ratio: 0.87, 95% confidence interval: 0.78–0.97).

**Conclusion:**

Falls and fear of falling were frequent in HD. High doses of neuroleptics, chorea, cognitive and behavioral symptoms, and particularly balance disorders contribute to falls in HD.

Falls are associated with impaired quality of life, fear of falling, and injury in Huntington's disease (HD).^1^ This is particularly important because, in HD, the degenerative process affects structures involved in locomotion, balance control, and cognition,^2^ thereby providing an anatomical substrate for the occurrence of falls throughout all stages of the disease^3^ and further contributing to the morbidity associated with this devastating condition. One multicenter study that included 158 individuals found that the fall prevalence over the past 30 days was 28.8%.^4^ Other studies demonstrated that for the preceding 6 months 22% to 38% of participants reported 1 fall and 36% reported multiple falls.^5^, ^6^ For the preceding 12 months, single falls rates were 21% to 75% and multiple falls 58% to 60%.^7^, ^8^ A few studies have used different clinical instruments, including Unified Huntington's Disease Rating Scale (UHDRS), kinematic gait analysis, mobility, and balance tests, to evaluate the potential contributors to falls in HD. They have postulated that motor symptoms like chorea, gait disturbance, and cognitive decline may contribute to occurrence of falls.^3^, ^7^ Fear of falling is common in HD patients (47%) and their caregivers (63.5%), as demonstrated in a multicenter study.^4^ There is limited data on injurious falls in HD. In 1 study, 73% were minor injuries like bruises and abrasions.^7^ The aims of this study are to describe the characteristics of falls in individuals with HD, compare the clinical characteristics of recurrent fallers and nonfallers, and investigate the relative contributions of different factors that may increase the risk of falls in HD. We hypothesized that clinical factors, as well as motor, cognitive, and behavioral symptoms and balance disorders, are potential contributors.

## Patients and Methods

### Participants

This was a single‐center, cross‐sectional, analytical observational study. We included consecutive patients with genetically confirmed (≥36) symptomatic HD attending the UFMG Chorea Clinic who provided informed consent. The exclusion criteria were a diagnosis of concurrent neurological disorders (stroke, epilepsy, neuropathy, and hereditary or acquired cerebellar ataxia), orthopedics issues such as fractures or symptomatic arthrosis, and severe visual, physical, or cognitive impairment preventing them from following the study protocol. The local ethical committee approved the study.

### Clinical Assessment

The scales and questionnaires were applied in the presence of care partners whenever possible. Clinical and demographic data were obtained from medical records. We converted the neuroleptic doses to olanzapine equivalents (OE).^9^ The definition of fall given to the participants was any unintended descent in which the participant came to rest on a lower level—whether the ground, floor, or another object—independent of the occurrence of injury. The history of falls was obtained using an adapted standardized questionnaire.^10^ To minimize recall bias, the account of falls was restricted to the 6 months prior to the evaluation.^10^ Individuals were classified as fallers if they reported ≥2 falls in the past 6 months, whereas the remaining were considered nonfallers.^10^ The participants were also asked to describe their most recent fall, and this information was used to classify the falls and analyze their circumstances. Falls were classified as “intrinsic falls” if caused by mobility and balance disorder or loss of consciousness, as “extrinsic” if caused by environmental factors (eg, collision), as “nonbipedal” (eg, fall from the bed), and as “unclassified.”^11^ We used the Falls Efficacy Scale‐International‐Brazil (FES‐I‐Brazil)^12^ to investigate the fear of falling. All patients were assessed using the Brazilian Portuguese versions of the Unified Huntington's Disease Rating Scale‐Total Motor Score (UHDRS‐TMS),^13^ the third part of the Unified Parkinson Disease Rating Scale (UPDRS, Part III),^14^ the Berg Balance Scale (BBS),^15^ the Timed Up and Go Test (TUG),^16^ and the Freezing of Gait Questionnaire (FOG‐Q).^17^ Due to the absence of a specific HD‐specific instrument, freezing was assessed using the FOG‐Q. The UPDRS, Part III, was applied as a complementary motor assessment due to its broader item‐level coverage of parkinsonian features compared to the UHDRS‐TMS. As parkinsonian features become progressively more clinically significant in later stages of HD,^18^ this broader coverage allows a more detailed quantification of the hypokinetic‐rigid component potentially relevant to fall risk.

We applied the pull test of the Movement Disorder Society‐Sponsored Revision of the Unified Parkinson's Disease Rating Scale (MDS‐UPDRS) without prior notice to simulate the unexpected falls that may occur in daily life and prevent learning effects that could interfere with the results.^19^ The investigation of cognitive and behavioral issues potentially associated with increased fall risk included the Brazilian Portuguese versions of the Frontal Assessment Battery (FAB),^20^ the Five Digit Test (FDT),^21^ the Hayling Test,^22^ the Ekman Emotion Facial Recognition Test,^23^ the Beck Depression Inventory‐II (BDI‐II),^24^ the Barratt Impulsiveness Scale (BIS‐11),^25^ the Neuropsychiatry Inventory‐Questionnaire (NPI‐Q),^26^ and the Irritability Scale.^27^

Spatiotemporal gait parameters were obtained during the 10‐Meter Walk Test.^28^ Refer to the Supporting Information for further details. The choice of those spatiotemporal parameters was based on previous studies.^3^

### Statistical Analysis

Descriptive statistics and tests for normality (Shapiro–Wilk) were calculated for all continuous outcomes. Baseline differences between “recurrent fallers” and “nonfallers” were analyzed using parametric (*t* test) or nonparametric tests (Mann–Whitney test and χ^2^ test). We used Pearson's correlation coefficient for parametric data and Spearman's rank test for nonparametric data to search for correlations. Additionally, stepwise forward logistic regression was performed to determine the variable related to recurrent falls. Model robustness was assessed using events‐per‐variable ratio, post hoc power for the observed effect, bootstrap internal validation (2000 resamples), and a sensitivity analysis to estimate the minimum detectable odds ratio (OR) at 80% power. At this point of the research, we determined that it is most important to avoid type II errors and did not apply Bonferroni correction. Statistical analysis was performed using IBM SPSS Statistics Macintosh, version 28.0.

## Results

Fifty‐eight patients who fulfilled the study criteria were invited to participate in the study. Eighteen patients refused, 40 individuals provided written informed consent and were interviewed. Of the latter, 24 reported having ≥2 falls in the past 6 months and, therefore, were considered recurrent fallers. The remaining individuals denied falling during the same period, except 1 individual who reported 1 fall and was considered nonfaller.

### Comparative Analyses of Clinical Characteristics

There were no significant differences in sex, age, age at disease onset, disease duration in years, or mean size of the CAG expansion between the 2 groups. In contrast, the OE was higher in the recurrent fallers group than in the nonfallers group (Table 1).

**TABLE 1:** Clinical characteristics

	Total sample (n = 40)	Value	Recurrent fallers (n = 24)	Nonfallers (n = 16)	*P*‐value
Sex (woman)	22 (55%)	n (%)	14 (58%)	8 (50%)	0.604
Age (years)	48.7 (13.82)	Mean (SD)	45.79 (14.27)	53.19	0.098
Age of disease onset (years)	42.30 (12.67)	Mean (SD)	39 (12.98)	46.56	0.082
Initial symptom					0.792
Motor	24 (60%)	n (%)	14 (58%)	10 (62.5%)	
Neuropsychiatric	40 (16%)	n (%)	10 (42%)	6 (37.5%)	
Disease duration (years)	5.92 (3.43)	n (%)	5.6 (3.65)	6.4 (3.22)	0.494
CAG repeat expansion size	44.64 (5.28)	Median (SD)	44 (5.99)	42.5 (11.27)	0.294
Use of medications					
Neuroleptics	32 (80%)	n (%)	20 (83.3%)	12 (75%)	0.519
Antidepressants	28 (70%)	n (%)	19 (79%)	9 (56.25%)	0.121
Anticonvulsants	3 (7.5%)	n (%)	3 (12.5%)	0	0.141
Benzodiazepines	1 (2.5%)	n (%)	1 (4.16%)	0	0.408
Antiparkinsonians	8 (20%)	n (%)	6 (25%)	2 (12.5%)	0.333
VMAT2 inhibitors	0	–	–	–	–
Olanzapine equivalents (mg/day)	8.29 (5.61)	Mean (SD)	10.0 (6.41)	5.85 (3.03)	0.028*

Abbreviations: VMAT2, vesicular monoamine transporter 2.

* *P* < 0.05.

### Comparative Analysis of Motor Symptoms, Balance, and Gait

The total UHDRS‐TMS scores were higher in the recurrent fallers group. The UHDRS subitem total chorea score (sum of all chorea scores), gait and postural instability, and UPDRS, Part III, were significantly higher in the recurrent fallers group, indicating more severe motor abnormalities than in the nonfallers group. The recurrent fallers group also had the worst results on the BBS. There was no significant difference between the 2 groups in TUG performance, spatiotemporal gait parameters, and FOG‐Q scores between the groups (Table 2). Eight participants endorsed feeling their feet stuck to the floor (FOG‐Q item 3 ≥1). Of these, 7 (87.5%) also reported start hesitation (item 5 ≥1) and turning hesitation (item 6 ≥1), supporting a consistent freezing phenotype, and 6 (75%) were recurrent fallers.

**TABLE 2:** Characteristics of motor symptoms, balance, and gait

	Value	Total sample (n = 40)	Recurrent fallers (n = 24)	Nonfallers (n = 16)	*P‐*value
UHDRS‐TMS	Median (minimum–maximum)	42.5 (4–72)	45.5 (19–72)	38 (4–59)	0.044*
Chorea total (sum of all UHDRS chorea scores)	Median (minimum–maximum)	8 (1–25)	11.5 (1–25)	7.5 (1–14)	0.020*
Gait	Median (minimum–maximum)	1 (0–2)	1 (0–3)	1 (0–2)	0.036*
Postural instability	Median (minimum–maximum)	1 (0–3)	2 (0–3)	0 (0–2)	0.001*
UPDRS, Part III, total	Median (minimum–maximum)	28.5 (1–67)	31.5 (6–67)	20 (1–53)	0.048*
Bradykinesia total	Median (minimum–maximum)	11.5 (1–43)	22 (7–43)	15.5 (1–36)	0.113
Rigidity total	Median (minimum–maximum)	0 (0–13)	1 (0–13)	1 (0–8)	0.389
BERG total	Median (minimum–maximum)	47 (22–56)	42.5 (22–56)	52 (30–56)	0.003*
Reaching forward	Median (minimum–maximum)	3 (0–4)	2 (0–4)	3 (1–4)	0.027*
Turn 360°	Median (minimum–maximum)	4 (1–4)	3 (1–4)	4 (1–4)	0.036*
Placing alternate foot on step	Median (minimum–maximum)	4 (0–4)	3 (0–4)	4 (1–4)	0.025*
Standing unsupported one foot in front	Median (minimum–maximum)	2 (0–4)	0.5 (0–4)	3 (0–4)	0.027*
Stading on one leg	Median (minimum–maximum)	1 (0–4)	0 (0–4)	2.5 (0–4)	0.006*
TUG	Mean (SD)	3.11 (3.27)	13.73 (3.50)	12.19 (2.73)	0.148
Step width (M)	Mean (SD)	9.92 (4.15)	9.87 (4.04)	9.98 (4.45)	0.859
Step width (CVM)	Mean (SD)	55.72 (38.71)	59.45 (37.76)	50.14 (40.67)	0.464
10MWT—preferred walking speed (m/s)	Mean (SD)	.83 (.20)	0.79 (0.18)	0.87 (0.81)	0.229
10MWT—maximum walking speed (m/s)	Mean (SD)	1.20 (.33)	1.16 (0.33)	1.26 (0.30)	0.165
Cadence	Mean (SD)	48.40 (9.33)	48.57 (10.61)	48.40 (7.40)	0.956
Step length (M)	Mean (SD)	49.77 (10.58)	48.81 (10.72)	51.20 (10.54)	0.492
Step length (CVM)	Mean (SD)	14.96 (8.51)	15.44 (9.51)	14.25 (6.96)	0.670
FOG total	Median (minimum–maximum)	3 (0–12)	4 (0–12)	0.5 (0–10)	0.132

* *P* < 0.05.

Abbreviations: BBS, Berg Balance Scale (↑ = better); CV, coefficient of variability; FOG‐Q, Freezing of Gait Questionnaire (↑ = worse); M, mean; 10MWT, 10‐Meter Walk Test; SD, standard deviation; TMS, total motor score; TUG, Timed Up and Go Test; UHDRS, Unified Huntington's Disease Rating Scale (↑ = worse); UPDRS, Unified Parkinson's Disease Rating Scale (↑ = worse).

### Comparative Analysis of Cognitive and Neuropsychiatric Symptoms

The times of choice and alternation of the FDT test were shorter in the non‐fallers group, indicating better performance in this test. There was a significant difference in the aberrant motor behavior subitems of the NPI, indicating that recurrent fallers presented this pattern of behavior more frequently than nonfallers. There was no significant difference in the performance on the FAB, Hayling Test, Ekman Test, BDI, Irritability Scale, and BIS‐11 between the groups **(**Table 3).

**TABLE 3:** Characteristics of cognitive and neuropsychiatric symptoms

	Value	Total sample (n = 40)	Recurrent fallers (n = 24)	Nonfallers (n = 16)	*P*‐value
Ekman—total (errors)	Mean (SD)	14.73 (5.16)	14.42 (5.11)	15.19 (5.36)	0.650
Irritability Scale	Mean (SD)	97.88 (6.69)	98.54 (5.32)	96.88 (8.45)	0.448
BIS‐11	Mean (SD)	60.00 (8.33)	60.54 (8.62)	59.19 (8.08)	0.621
FAB	Mean (SD)	7.78 (4.01)	7.5 (4.00)	8.19 (4.35)	0.610
FDT—reading (time)	Median	0:01:25.00	00:01:15.00	0:01:06.99	0.062
FDT—reading (errors)	Median	0.00	0.00	0.00	0.733
FDT—counting (time)	Median	0:01:32.00	0:01:21.99	0:01:17.00	0.202
FDT—counting (errors)	Median	0.00	1.00	0.00	0.521
FDT—choosing (time)	Median	0:02:02.00	0:02:16.50	0:01:46.50	0.023*
FDT—choosing (errors)	Median	4.50	6.00	0.00	0.521
FDT—shifting (time)	Median	02.41:00	0:03:07.00	0:02:27.50	0.020*
FDT—shifting (errors)	Median	4.50	3.00	1.00	0.795
Hayling, part A (time)	Median	0:00:35.03	15.00	15.00	0.521
Hayling, part A (errors)	Median	14.00	0:00:30.79	0:02:58.63	0.895
Hayling, part B (time)	Median	01:17.90	0:01:46.39	0:01:46.39	0.149
Hayling, part B (errors)	Median	6.00	6.00	13.00	0.817
BDI‐II	Mean (SD)	16.63 (13.27)	10.00	0.00	0.058
NPI‐Q—total	Mean (SD)	17.03 (14.83)	24 (20.33)	12.75 (11.65)	0.093
Aberrant motor	Mean (SD)	1.68 (3.74)	2.58 (4.32)	0.31 (0.87)	0.013*

* *P* < 0.05.

Abbreviations: BDI‐II, Beck Depression Inventory‐II (depressive symptoms); BIS‐11, Barratt Impulsiveness Scale (impulsivity); Ekman, 60‐Faces Test (social cognition); FAB, Frontal Assessment Battery (global executive/frontal‐lobe function); FDT, Five Digit Test (processing speed and executive function); Hayling Test (executive function—response initiation and inhibition); Irritability Scale (irritability); NPI‐Q, Neuropsychiatry Inventory‐Questionnaire (neuropsychiatric symptoms); SD, standard deviation.

### Falls Questionnaire and Fear of Falling

We collected data of 25 falls (Table 4). The Falls Questionnaire has shown that fallers (79%) and nonfallers (75%) reported high rates of fear of falling. Both groups have also demonstrated high scores on FES‐I, indicating high levels of fear of falling. There was no statistical difference between the groups regarding fear of falling. Restrictions in daily activities because of fear of falling and problems with multiple tasks were also frequently reported in both groups. The most common situations during which falls occurred were walking, having a shower, and doing housework. Only 1 patient reported a premonitory feeling before falling. Thirty‐six percent of falls occurred while multitasking. Sixty‐four percent of the fallers reported injury because of falls, but they were all mild, with the exception of 1 patient who reported a fracture. Most falls were classified as “intrinsic” (Table 5).

**TABLE 4:** Falls Questionnaire, FES‐I

	Value	Total sample (n = 40)	Recurrent fallers 24 (60%)	Nonrecurrent 16 (40%)	*P‐*value
Number of falls (≤ 6 months)	Mean (SD)	2.45 (2.63)	4 ± 2.33	0.13 ± 0.342	>0.001*
Fear of falling	n (%)	40 (77.5%)	19 (79.17%)	12 (75%)	0.757
Restriction of daily activities because fear of falling	n (%)	40 (52.5%)	14 (58.33%)	7 (43.75%)	0.373
Problems with multiple tasks	n (%)	40 (82.5%)	22 (91%)	11 (68%)	0.062
FES‐I	Mean (SD)	31.95 (12.68)	34.71 ± 13.68	27.81 ± 10.02	0.074

* *P* < 0.05.

Abbreviations: FES‐I, Falls Efficacy Scale‐International; SD, standard deviation.

**TABLE 5:** Most frequently reported characteristics, circumstances, and consequences of falls

Indoor	18 (72%)
Activity at the time of fall	
Walking	10 (40%)
Housework	3 (12%)
Showering	4 (16%)
Felt something before falling	1 (4%)
Performing 2 simultaneous activities when fell	9 (36%)
Injury	16 (64%)
Kind of injury	
Scratch	8 (32%)
Laceration	4 (16%)
Bruise	3 (12%)
Falls classification	
Intrinsic	19 (76%)
Extrinsic	4 (16%)
Nonbipedal	2 (8%)

### Correlations and Logistic Regression

Moderately positive correlations were observed between OE and chorea (ρ = 0.631, *P* < 0.001) and step length variability (ρ = 0.688, *P* < 0.001) in the recurrent fallers group. In the same group, moderately negative correlations were observed between OE and BBS (ρ = −0.472, *P* < 0.002) and step length (ρ = −0.517, *P* < 0.001). In the whole sample, OE was moderately and significantly correlated with the total UPDRS, Part III, score (ρ = 0.44, *P* = 0.017). FOG‐Q total scores correlated with overall motor severity (UHDRS‐TMS, ρ = 0.68, *P* < 0.001) and, to a lesser extent, with chorea severity (ρ = 0.47, *P* = 0.002). Logistic regression was performed to assess whether neuroleptic dose, total chorea score, BBS, and FDT alternation time could be predictors of recurrent falls in HD. The final model showed an events‐per‐variable ratio of 16, a post hoc power of 70%, and bootstrap stability (median OR: 0.86, 95% confidence interval [CI]: 0.68–0.95; BBS retained in 83% of resamples); sensitivity analysis showed 80% power to detect ORs ≤0.91 per BBS point.

## Discussion

For the preceding 6 months our multiple falls rate (60%) is higher than previously reported by Kloos et al. (38%).^6^ A higher fall rate in our cohort is plausibly explained by sample‐specific characteristics: a tertiary‐care HD clinic with predominantly symptomatic patients, moderate‐to‐advanced motor impairment, and high neuroleptic exposure—a well‐established fall‐risk factor.^29^ Other studies have also shown that approximately 60% of patients were considered recurrent fallers, although they used different criteria (≥2 falls in the preceding 12 months).^7^, ^8^ One of those studies with a 3‐month follow‐up using a standardized calendar showed an incidence of 20% for ≥2 falls and 40% for 1 fall in the period. The short follow‐up period and underreporting possibly explain the decrease in fall incidence in the prospective phase of the study.^7^ As already demonstrated in Parkinson's disease (PD), prospective evaluations and standardized questionnaires have revealed a considerably higher incidence of falls than in retrospective studies.^10^

Falls were classified as “intrinsic falls” (76%), which frequently occurred indoors during walking (40%) and multitasking (12%), indicating disturbance of locomotor and cognitive systems as contributing factors. We also found high rates of adverse fall consequences, although fortunately these were mainly soft tissue damage. This might be explained by the young age of our cohort and high occurrence of falls indoor, where house furniture such as sofas and carpets may prevent physical injury. In our study, both groups reported high rates of fear of falling, and there was no difference in fear of falling between the groups. Moreover, there were restrictions on daily activities because of fear of falling as reported in the Falls Questionnaire. Low perceived self‐efficacy in avoiding falls during essential activities was reported by both groups (FES‐I). The use of FES‐I, a multidimensional scale capturing concerns about falling rather than the binary occurrence of past falls, may also have contributed to detecting fear in both groups. Awareness of disease progression, fall risk, and family experience with advanced HD likely converges to elevated fear of falling regardless of fall history. Our results differ from those of another study where low rates of fear of falling were reported in the fallers group despite patients demonstrating low balance confidence.^7^ A multicenter study assessing 158 individuals concluded that fear of falling is common in HD patients (47%) and their formal (25%) and informal caregivers (63%). They reported that anticipatory awareness of fall risk and gender are predictors of fear of falling in HD. Moreover, in the multivariable analysis recent falls were not an independent predictor of fear of falling in either care‐independent (OR: 1.09, *P* = 0.77) or care‐dependent (OR: 0.98, *P* = 0.92) subjects.^4^ This is consistent with our findings and previous observations that fear of falling is independent of actual falls,^30^ suggesting that fear of falling in HD is not merely a reactive consequence of having fallen and may therefore warrant intervention independently of fall history. Therefore, we believe that early physical therapy and exercise interventions,^31^ environmental adaptation, and anxiety reduction^4^ may be applied to HD to attenuate fear of falling.

Regarding the comparative analysis of the clinical characteristics of recurrent fallers and nonfallers, there was no significant difference in sex, age, CAG repeat expansion size, age at disease onset, and use of medications (Table 1). However, the OE was significantly higher in the fallers group. The use of neuroleptics, principally haloperidol, risperidone, and olanzapine, commonly administered at high doses and for an extended period in HD, has been proven to cause impairment in postural control^32^ and drug‐induced parkinsonism.^33^ In the whole sample, OE was moderately and significantly correlated with the total UPDRS, Part III, score. When stratified by faller status, the correlation with UPDRS, Part III, did not reach significance in either subgroup likely reflecting reduced statistical power. There were no correlations between OE and bradykinesia (sum of all bradykinesia scores of UPDRS), rigidity (sum of all rigidity scores of UPDRS), or gait velocity in the recurrent fallers group. The UPDRS, Part III, score was higher in the fallers group. In addition, Grimbergen et al. reported that recurrent fallers performed worse in bradykinesia evaluating the subitems of UHDRS.^7^ A large number of biomechanical studies demonstrate that bradykinesia is present in patients with HD, even in the early to middle phases.^3^ Therefore, the current evidence remains inconclusive as to whether parkinsonism—either HD related or drug induced—contributes to the risk of falls in HD, and further studies are needed to determine this potential association. We found a moderately negative correlation of OE with BBS and step length in the recurrent fallers group. Moreover, in the recurrent fallers group, we found a moderately positive correlation of OE with UHDRS‐TMS, step length variability, and chorea total (sum of all scores evaluating chorea in the UHDRS‐TMS). However, we did not find a correlation between OE and any of those variables in the nonfallers group. These findings suggest that high neuroleptic doses might contribute to falls in HD through balance disturbance rather than drug‐induced parkinsonism. The higher chorea score suggests that neuroleptics were used more in the fallers to control the more severe hyperkinesia. It is also possible that patients who require higher doses of neuroleptics have more severe disease with neuropathological damage of balance control centers. The presence of higher values of chorea scores and UHDRS‐TMS, findings of another study as well,^7^ supports this hypothesis. It is well established that the UHDRS‐TMS is an appropriate measure of motor symptom severity in HD, and it has been negatively correlated with functional capacity.^18^ We also believe that choreic movements that exceed the limits of postural control contribute to the occurrence of falls during HD. Furthermore, sedation may be a contributing factor to falls in patients using high‐dosage neuroleptics.

Differences were also significant in the gait evaluation subitems of the UHDRS, indicating that recurrent fallers had worse performance. Although no statistically significant differences were detected in quantitative gait parameters, numerical differences in the expected direction were observed (lower speed, shorter step length and greater step length, and step width variability) among recurrent fallers. Due to the modest sample size, type II error cannot be excluded, and these findings should be interpreted with caution. A previous study using dual‐task paradigms in HD similarly failed to find correlation between gait parameters and fall history.^34^ In contrast, previous studies have shown that recurrent fallers exhibit decreased gait velocity,^7^, ^8^ stride length, mediolateral trunk sway, and increased variability in stride length.^7^ Our results must be interpreted with caution, because it is possible that the small sample size and heterogeneity of our cohort may have affected the analysis.

The occurrence of FOG in HD is rarely reported in literature. The presence of FOG in the video of 1 patient in a case report, especially while turning, is the only mention of FOG in HD‐related gait disorders.^35^ We included the FOG‐Q because of our observation of this phenomenon in a few patients and its possible association with falls. However, we did not find differences between the groups although 8 participants described phenomena consistent with FOG. These findings suggest that FOG is an overlooked finding in HD.

We found no significant difference in the TUG performance between the 2 groups. This result differs from a previous study, where a cutoff of <14 seconds was associated with an increased risk of fall in HD.^8^ Our finding aligns, however, with more recent and larger studies. Youhanan et al. reported that the TUG did not differentiate fallers from nonfallers (*P* = 0.098) and showed only moderate discriminative ability (area under the curve = 0.66), with an optimal cutoff of 11.5 seconds—well below the previously proposed 14‐second threshold.^36^ Similarly, Kaur et al. found that TUG values cluster tightly across the Huntington Disease Integrated Staging System (HD‐ISS), with <2 seconds separating healthy controls from Stage 3 patients, indicating limited sensitivity to disease progression.^37^ As an aggregate measure of sit to stand, gait, and turning, the TUG may be confounded by chorea and high gait variability in HD, and it has not been formally validated in this population.^18^ A single, universal cutoff is therefore unlikely to generalize across cohorts with different stages, severities, and treatment exposures. Future research should explore stage‐stratified or disease‐specific cutoffs—for example, anchored to the HD‐ISS or total functional capacity—and clinical practice may benefit from combining the TUG with additional balance and cognitive measures rather than relying on it as a stand‐alone fall risk screening tool in HD.

For a more comprehensive balance approach, we used the BBS and found significant differences between the groups, indicating that fallers performed worse. Deficits in dynamic balance items likely reflect compromise of automatic postural reflexes and integration of cognitive resources required for balance recovery—both progressively impaired in HD.^2^ Higher chorea severity, neuroleptic exposure, and executive dysfunction may further contribute to worse BBS performance in fallers. Therefore, previous studies have shown that the BBS may be useful for fall risk assessment^8^ and detection of balance disturbance in HD^38^ even in the pre‐manifest stage.^39^

The dissociation between a significant BBS finding and nonsignificant TUG, gait, and FOG comparisons likely reflects the multidimensional nature of fall risk in HD: the BBS aggregates static and dynamic balance items and integrates motor and cognitive components, whereas the TUG (an aggregated time) and isolated gait or FOG measures may dilute the relevant signal in a heterogeneous cohort. This argues against relying on a single instrument for fall risk screening in HD.

Regarding the comparative analysis of cognitive disturbances between the groups, we found a significant difference in the times of choice and alternation of FDT, indicating that recurrent fallers had more executive cognitive dysfunction. Another study has assessed cognition deficits and fall risk in HD, also indicating an association between executive deficits and increased fall risk.^7^ Beyond statistical association, the link between executive dysfunction and falls in HD likely reflects a shared fronto‐striatal substrate, in which striatal degeneration disrupts both automatic postural control and the attentional resources required to recover from perturbations.^40^ Consistent with this, dual‐task walking deficits have been shown to correlate with prospective falls in early‐ to mid‐stage HD.^41^ Patients exhibit disproportionate increases in postural sway under cognitive load,^42^ suggesting reliance on a “posture‐second” strategy^43^ that becomes maladaptive when executive reserve is reduced. This mechanistic framework aligns with our finding that 36% of falls occurred during multitasking.

Due to the exploratory nature of our study, we included tools to investigate possible associations of different cognitive and behavioral domains with the occurrence of falls in HD. However, as Table 3 indicates, there were no other significant differences in the comparison between the groups, except the aberrant motor behavior subitem of the NPI, where recurrent fallers had more severe symptoms than nonfallers. The possible occurrence of wandering and repetitive motor behavior can increase the likelihood of falls, such as repeatedly taking off and putting on clothes. Only 1 additional study evaluating behavior and fall risk in HD indicates the association between aggressiveness and a higher risk of falling.^7^ The roles of cognitive and neuropsychiatric disorders in falls in HD remain largely unexplored. Nevertheless, considering the evidence presented here, behavioral and principally cognitive disorders increase the fall risk in HD.

To identify the possible predictors of falls in HD, we performed logistic regression, including neuroleptic dose, total chorea (sum of all UHDRS chorea scores), total BBS, and FDT alternation time. The regression analysis revealed that only the BBS scores were retained in the model (OR: 0.87, 95% CI: 0.78–0.97), indicating that measures of postural instability can predict the occurrence of falls in HD. This finding has practical implications for both clinical care and research. Clinically, the BBS is a brief, low‐cost, equipment‐free instrument that could be incorporated into routine HD outpatient follow‐up to identify patients at increased fall risk, including those without a previous fall history, and to guide timely referral to balance‐focused rehabilitation. For research, our results support the BBS as a candidate outcome measure in fall‐prevention trials in HD and highlight the need for prospective studies to confirm its predictive validity.

The present study has some limitations such as small sample size, reliance on retrospective information provided by subjects and care partners, and the absence of staging and systematic rehabilitation/exercise data collection.

In conclusion, falls occur frequently in HD and have physical and emotional consequences as high rates of fear of falling were reported in both groups. Our study points out that the risk of falls in HD is multifactorial, suggesting that chorea, high doses of neuroleptics, cognitive and behavioral symptoms, and particularly balance disorders must contribute to their occurrence. Future studies should confirm these findings in prospective, longitudinal designs, ideally stratified by disease stage (eg, HD‐ISS) and combined with objective digital tools—such as wearable inertial sensors or instrumented posturography—to improve sensitivity and explore multidimensional risk‐prediction models specific to HD.

## Author Roles

A.P.F.: design, execution, analysis, writing, editing; F.E.C.C.: design, execution, analysis, editing); S.E.S.A.L.: execution; A.A.S.: design, analysis, editing; R.O.H.M.: design; D.P.M.: design.

## Disclosures

**Ethical Compliance Statement:** This study was conducted in accordance with the ethical standards of the institutional and national research committee and with the 1964 Helsinki Declaration and its later amendments. The research protocol was approved by the Research Ethics Committee of the Universidade Federal de Minas Gerais (UFMG), Brazil, through Plataforma Brasil (approval number: 2725403; CAAE: 84713618.0.00005149). Written informed consent was obtained from all participants prior to their inclusion in the study. We confirm that we have read the journal's position on issues involved in ethical publication and affirm that this work is consistent with those guidelines.

## Financial Disclosures and Conflicts of Interest

Author disclosures are available in the Supporting Information.

## Supporting information


**Data S1**. Detailed protocols of the clinical instruments used to assess gait, balance, and postural instability—equipment, application procedures, scoring, and validation references for the 10‐Meter Walk Test (10MWT), the Berg Balance Scale (BBS), the Timed Up and Go Test (TUG), the Freezing of Gait Questionnaire (FOG‐Q), and the pull test (MDS‐UPDRS [Movement Disorder Society‐Sponsored Revision of the Unified Parkinson's Disease Rating Scale], item 3.12) (Microsoft Word file).

**Data S2.** COI_disclosure.

## Data Availability

The data that support the findings of this study are available from the corresponding author upon reasonable request.

## References

[1] Homann CN , Homann B , Ivanic G , Urbanic‐Purkart T . Accidental falls in patients with hyperkinetic movement disorders: a systematic review. Tremor Other Hyperkinet Mov (N Y) 2022;12:30. 10.5334/tohm.709. 36303814 PMC9541119

[2] Rüb U , Seidel K , Heinsen H , Vonsattel JP , den Dunnen WF , Korf HW . Huntington's disease (HD): the neuropathology of a multisystem neurodegenerative disorder of the human brain. Brain Pathol 2016;26(6):726–740. 10.1111/bpa.12426. 27529157 PMC8029421

[3] Vuong K , Canning CG , Menant JC , Loy CT . Gait, balance, and falls in Huntington disease. Handb Clin Neurol 2018;159:251–260. 10.1016/B978-0-444-63916-5.00016-1. 30482318

[4] Kalkers K , Schols JMGA , van Zwet EW , Roos RAC . Falls, fear of falling, and preventive measures in Huntington's disease: the perspectives of individuals with Huntington's disease and caregivers in long‐term care. J Huntingtons Dis 2021;10(4):493–503. 10.3233/JHD-210493. 34719503

[5] Kloos AD , Kegelmeyer DA , Young GS , Kostyk SK . Fall risk assessment using the Tinetti mobility test in individuals with Huntington's disease. Mov Disord 2010;25(16):2838–2844. 10.1002/mds.23421. 20960478

[6] Kloos AD , Kegelmeyer DA , White SE , Kostyk SK . The impact of different types of assistive devices on gait measures and safety in Huntington's disease. PLoS One 2012;7(2):e30903. 10.1371/journal.pone.0030903. 22363511 PMC3281896

[7] Grimbergen Y , Knol M , Bloem B , Kremer B , Roos R , Munneke M . Falls and gait disturbances in Huntington's disease. Mov Disord 2008;23:970–976. 18381643 10.1002/mds.22003

[8] Busse M , Wiles C , Rosser A . Mobility and falls in people with Huntington's disease. J Neurol Neurosurg Psychiatry 2009;80:88–90. 19091714 10.1136/jnnp.2008.147793

[9] Leucht S , Samara M , Heres S , et al. Dose equivalents for second‐generation antipsychotic drugs: the classical mean dose method. Schizophr Bull 2015;41:1397–1402. 25841041 10.1093/schbul/sbv037 PMC4601707

[10] Bloem BR , Grimbergen YA , Cramer M , Willemsen M , Zwinderman AH . Prospective assessment of falls in Parkinson's disease. J Neurol 2001;248:950–958. 11757958 10.1007/s004150170047

[11] Lach HW , Reed AT , Arfken CL , et al. Falls in the elderly: reliability of a classification system. J Am Geriatr Soc 1991;39:197–202. 1991951 10.1111/j.1532-5415.1991.tb01626.x

[12] Flávia FOC . Adaptação transcultural e avaliação das propriedades psicométricas da Falls Efficacy Scale ‐ International em idosos brasileiros. Rev Bras Fisioter 2010;14:237–243. 20730369

[13] Tumas V , Camargos ST , Jalali PS , Galesso AP , Marques W Jr . Internal consistency of a Brazilian version of the unified Huntington's disease rating scale. Arq Neuropsiquiatr 2004;62:977–982. 15608955 10.1590/S0004-282X2004000600009

[14] Fahn S , Elton RL , UPDRS Development Committee . The unified Parkinson's disease rating scale. In: Fahn S , Marsden CD , Calne DB , Goldstein M , eds. Recent Developments in Parkinson's Disease. 2nd ed. Florham Park: McMellam Health Care Information; 1987:p153–p163.

[15] Miyamoto ST , Lombardi Júnior I , Berg KO , Ramos LR , Natour J . Brazilian version of the Berg balance scale. Braz J Med Biol Res 2004;37:1411–1421. 15334208 10.1590/s0100-879x2004000900017

[16] Faria C , Teixeira‐Salmela L , de Araújo P , Polese J , Nascimento L , Nadeau S . TUG‐ABS Portuguese‐Brazil: a clinical instrument to assess mobility of hemiparetic subjects due to stroke. Rev Neurocienc 2015;23:357–367.

[17] Baggio JAO , Curtalli MB , Rodrigues GR , Tumas V . Validação da versão brasileira da escala de congelamento da marcha. Arq Neuropsiquiatr 2012;70:599–603.22899031

[18] Mestre TA , Forjaz MJ , Mahlknecht P , et al. Rating scales for motor symptoms and signs in Huntington's disease: critique and recommendations. Mov Disord Clin Pract 2018;5:111–117. 30363393 10.1002/mdc3.12571 PMC6174417

[19] Bloem BR , Beckley DJ , van Hilten JJ , Roos RA . Clinimetrics of postural instability in Parkinson's disease. J Neurol 1998;245:669–673. 9776467 10.1007/s004150050265

[20] Beato R , Nitrini R , Formigoni AP , Caramelli P . Brazilian version of the frontal assessment battery (FAB): preliminary data on administration to healthy elderly. Dement Neuropsychol 2007;1:59–65. 29213369 10.1590/S1980-57642008DN10100010 PMC5619385

[21] de Paula JJ , Oliveira TD , Querino EHG , Malloy‐Diniz LF . The five digits test in the assessment of older adults with low formal education: construct validity and reliability in a Brazilian clinical sample. Trends Psychiatry Psychother 2017;39:173–179. 28876361 10.1590/2237-6089-2016-0060

[22] Zimmermann N , Cardoso CO , Kristensen CH , Fonseca RP . Brazilian norms and effects of age and education on the Hayling and trail making tests. Trends Psychiatry Psychother 2017;39:188–195. 28977074 10.1590/2237-6089-2016-0082

[23] Ekman P , Friesen WV . Constants across cultures in the face and emotions. J Pers Soc Psychol 1971;17:124–129. 5542557 10.1037/h0030377

[24] Gomes‐Oliveira MH , Gorenstein C , Neto FL , Andrade LH , Wang YP . Validation of the Brazilian Portuguese version of the Beck depression inventory‐II in a community sample. Rev Bras Psiquiatr 2012;34:389–394. 23429809 10.1016/j.rbp.2012.03.005

[25] Tavares H , Vasconcelos AG , Fuentes D . Traduçao e adaptaçao cultural da Barratt Impulsiveness Scale (BIS‐11) para aplicaçao em adultos brasileiros. J Bras Psiquiatr 2010;59:99–105.

[26] Camazzoto A , Godinho C , Kochhann A , MAssochini G , Chaves M . Validity of the Brazilian version of the neuropsychiatric inventory questionnaire (NPI‐Q). Arq Neuropsiquiatr 2015;73:41–45. 25608126 10.1590/0004-282X20140177

[27] Chatterjee A , Anderson KE , Moskowitz CB , Hauser WA , Marder KS . A comparison of self‐report and caregiver assessment of depression, apathy, and irritability in Huntington's disease. J Neuropsychiatry Clin Neurosci 2005;17:378–383. 16179661 10.1176/jnp.17.3.378

[28] Van Loo MA , Moseley AM , Bosman JM , de Bie RA , Hassett L . Inter‐rater reliability and concurrent validity of step length and step width measurement after traumatic brain injury. Disabil Rehabil 2003;25:1195–1200. 14578058 10.1080/09638280310001599989

[29] Seppala LJ , Wermelink AMAT , de Vries M , et al. Fall‐risk‐increasing drugs: a systematic review and meta‐analysis: II. Psychotropics. J Am Med Dir Assoc 2018;19(4):371.e11–371.e17. 10.1016/j.jamda.2017.12.098. 29402652

[30] Schoene D , Heller C , Aung YN , Sieber CC , Kemmler W , Freiberger E . A systematic review on the influence of fear of falling on quality of life in older people: is there a role for falls? Clin Interv Aging 2019;14:701–719. 31190764 10.2147/CIA.S197857 PMC6514257

[31] Fritz NE , Rao AK , Kegelmeyer D , et al. Physical therapy and exercise interventions in Huntington's disease: a mixed methods systematic review. J Huntingtons Dis 2017;6(3):217–235. 10.3233/JHD-170260. 28968244 PMC5676854

[32] de Groot MH , van Campen JP , Moek MA , Tulner LR , Beijnen JH , Lamoth CJ . The effects of fall‐risk‐increasing drugs on postural control: a literature review. Drugs Aging 2013;30:901–920. 24005984 10.1007/s40266-013-0113-9

[33] Shin HW , Chung SJ . Drug‐induced parkinsonism. J Clin Neurol 2012;8:15–21. 22523509 10.3988/jcn.2012.8.1.15 PMC3325428

[34] Purcell NL , Goldman JG , Ouyang B , Liu Y , Bernard B , O'Keefe JA . The effects of dual‐task cognitive interference on gait and turning in Huntington's disease. PLoS One 2020;15:e0226827. 31910203 10.1371/journal.pone.0226827 PMC6946131

[35] Termsarasab P , Frucht SJ . The “stutter‐step” gait: a peculiar gait feature in advanced Huntington's disease and chorea‐acanthocytosis. Mov Disord Clin Pract 2018;5:223–224. 30746406 10.1002/mdc3.12586 PMC6336370

[36] Youhanan N , Kaur J , Hall A , et al. Examining balance and the likelihood of falls in Huntington's disease. Clin Park Relat Disord 2025;13:100399. 10.1016/j.prdoa.2025.100399 Erratum in: Clin Park Relat Disord. 2025 Nov 22;13:100408. 41169989 PMC12569833

[37] Kaur J , Youhanan N , Bagga K , Hall A , Gilbert PE , Goble DJ , Corey‐Bloom J . Using a brief body sway assessment device to track balance differences across the Huntington's disease integrated staging system Spectrum. Mov Disord Clin Pract 2026;13(2):475–481. 10.1002/mdc3.70288. 40922521 PMC12911521

[38] Rao AK , Muratori L , Louis ED , Moskowitz CB , Marder KS . Clinical measurement of mobility and balance impairments in Huntington's disease: validity and responsiveness. Gait Posture 2009;29:433–436. 19111470 10.1016/j.gaitpost.2008.11.002

[39] Quinn L , Khalil H , Dawes H , et al. Outcome measures subgroup of the European Huntington's disease network. Reliability and minimal detectable change of physical performance measures in individuals with pre‐manifest and manifest Huntington disease. Phys Ther 2013;93:942–956. 23520147 10.2522/ptj.20130032

[40] McColgan P , Seunarine KK , Razi A , et al. Track‐HD investigators. Selective vulnerability of Rich Club brain regions is an organizational principle of structural connectivity loss in Huntington's disease. Brain 2015;138(Pt 11):3327–3344. 10.1093/brain/awv259. 26384928 PMC4620513

[41] Peters J , Lauinger A , Mayr M , Ginell K , Abou L . Dual‐task assessments for predicting future falls in neurologic conditions: a systematic review. Am J Phys Med Rehabil 2024;103(6):554–560. 10.1097/PHM.0000000000002452. 38466165

[42] Muratori LM , Quinn L , Li X , Youdan G , Busse M , Fritz NE . Measures of postural control and mobility during dual‐tasking as candidate markers of instability in Huntington's disease. Hum Mov Sci 2021;80:102881. 10.1016/j.humov.2021.102881. 34583142

[43] Yogev‐Seligmann G , Rotem‐Galili Y , Dickstein R , Giladi N , Hausdorff JM . Effects of explicit prioritization on dual task walking in patients with Parkinson's disease. Gait Posture 2012;35(4):641–646. 10.1016/j.gaitpost.2011.12.016. 22342204

