# Supplementary material for: Falls in Huntington's Disease: A Cross‐Sectional Analysis of Clinical Features and Potential Contributors
Source: Mov Disord Clin Pract. 2026 Jul 30:10.1002/mdc3.70754. Online ahead of print. doi: 10.1002/mdc3.70754 (PMC13424261; doi:10.1002/mdc3.70754)
Supplement: Supplementary file 1 — Data S1. Detailed protocols of the clinical instruments used to assess gait, balance, and postural instability—equipment, application procedures, scoring, and validation references for the 10‐Meter Walk Test (10MWT), the Berg Balance Scale (BBS), the Timed Up and Go Test (TUG), the Freezing of Gait Questionnaire (FOG‐Q), and the pull test (MDS‐UPDRS [Movement Disorder Society‐Sponsored Revision of the Unified Parkinson's Disease Rating Scale], item 3.12) (Microsoft Word file). [file MDC3-9999-0-s001.docx]

**Supplementary Material**

**Detailed protocols of the clinical instruments used to assess gait, balance, and postural instability**

This Supplementary Material describes in detail the procedures used to assess spatiotemporal gait parameters, balance, mobility, freezing of gait, and reactive postural control in the present study. All instruments were applied by trained examiners using the Brazilian Portuguese versions of the validated tools cited in the main manuscript. Each instrument is presented in a separate section, with information on equipment, application protocol, scoring, and the validation reference used.

**1. Ten-Meter Walk Test (10mWT)**

*Equipment*

Data collection required a flat, non-slippery 10-meter walkway, marked at both ends with adhesive tape and with an additional 2-meter acceleration and 2-meter deceleration zone at each end, resulting in a total walkway length of 14 meters. Two non-toxic ink pens, one per heel, were securely fastened to the posterior aspect of each shoe using adhesive tape, with the writing tip oriented perpendicular to the floor so that each heel strike produced a visible mark. The walkway was covered with a roll of white paper or, alternatively, with a smooth painted floor protected by removable adhesive sheets, in order to register heel marks across the full 10-meter path. A manual measuring tape with 0.1 cm precision was used for the offline measurement of step length and step width, and a digital stopwatch with 0.01 s precision was used for timing each trial.

*Participant preparation*

Participants wore their habitual flat footwear. The ink pens were taped to the participants' heels so that each heel strike left a mark on the floor as they walked along the 10-meter path. The examiner verified that the tip of each pen contacted the floor at the moment of heel strike without interfering with the natural gait pattern. Whenever a pen was found to be loose, displaced, or producing inconsistent marks, the trial was discarded and repeated.

*Walking trials*

Each participant performed two trials at preferred (self-selected, comfortable) walking speed and two trials at maximum (as fast as safely possible) walking speed, with at least 30 seconds of seated rest between trials to prevent fatigue. The participant was instructed to start walking from a static standing position located 2 meters before the start line and to continue walking for 2 meters beyond the end line, so that acceleration and deceleration phases were excluded from the analyzed 10-meter segment. The examiner walked slightly behind and to the side of the participant for safety, without providing pacing cues.

Timing started when the participant's leading foot crossed the start line and stopped when the same foot crossed the end line. The mean of the two trials at each speed was used in the analysis.

*Offline measurement of heel marks*

After each trial, and before the participant performed the next one, the examiner identified the heel marks corresponding to the central 10-meter segment of the walkway and labeled them sequentially as right (R) and left (L).

Step length was defined as the longitudinal distance between the heel mark of one foot and the heel mark of the contralateral foot at the next heel strike (e.g., from R_n_ to L_n_, and from L_n_ to R_n+1_). Measurements were taken in centimeters with a manual tape measure laid along the direction of progression. Step width was defined as the perpendicular (mediolateral) distance between the right and left heel marks of two consecutive heel strikes (e.g., the perpendicular distance from L_n_ to the line of progression defined by R_n_ and R_n+1_).

Whenever heel marks were ambiguous (e.g., smudged, overlapping, or missing), the corresponding step was excluded from the calculation. A minimum of eight valid consecutive steps per trial was required for the trial to be considered valid.

*Derived spatiotemporal parameters*

Cadence was calculated as the number of steps per minute, derived from the total number of heel strikes within the 10-meter segment and the time taken to traverse it, according to the formula cadence = (number of steps / time in seconds) x 60.

Gait velocity (m/s) was derived from the time taken to complete the 10-meter distance, computed separately for preferred and maximum walking speeds, according to the formula velocity = 10 / time in seconds. Values are reported as the mean of the two valid trials at each speed.

Variability was characterized by the coefficient of variation (CV), computed for step length and step width as CV (%) = (standard deviation / mean) x 100, using all valid consecutive steps recorded during the preferred-speed trials. A higher CV indicates greater stride-to-stride variability and has been associated with impaired locomotor control.

**2. Berg Balance Scale (BBS)**

*Overview*

The Berg Balance Scale is a 14-item performance-based instrument designed to assess static and dynamic balance during functional tasks of increasing difficulty. In the present study, we used the Brazilian Portuguese version validated by Miyamoto et al. (2004), which has demonstrated high inter- and intra-rater reliability in adult and elderly populations.

*Equipment*

Application of the scale required a chair with armrests (used as the starting position for most tasks), a second chair without armrests (used for transfers), a stopwatch, a ruler, a small object placed on the floor (used in the bending-forward task), and a step or low stool. A clear and unobstructed space approximately 3 meters wide was required to allow safe execution of standing and turning tasks.

*Application protocol*

Each of the 14 items was administered in the order specified by the original protocol: (1) sitting to standing, (2) standing unsupported, (3) sitting unsupported, (4) standing to sitting, (5) transfers between chairs, (6) standing with eyes closed, (7) standing with feet together, (8) reaching forward with outstretched arm while standing, (9) picking up an object from the floor, (10) turning to look behind, (11) turning 360 degrees, (12) placing alternate foot on a step or stool, (13) standing with one foot in front of the other, and (14) standing on one leg. Verbal instructions were standardized and demonstrated when necessary. Participants wore their habitual footwear, and an examiner remained close at all times to ensure safety, without providing physical or verbal support that could influence performance.

*Scoring*

Each item was scored on a 5-point ordinal scale ranging from 0 (unable to perform the task or requires maximal assistance) to 4 (independent and meeting all time and distance criteria). The total score ranges from 0 to 56 points, with higher values indicating better balance performance. Lower BBS scores have been consistently associated with increased fall risk in neurological populations, including Huntington's disease, and were therefore included as a candidate predictor in the logistic regression model of the main analysis.

**3. Timed Up and Go (TUG)**

*Overview*

The Timed Up and Go test is a brief, performance-based assessment of functional mobility that integrates components of strength, balance, gait, and transitional movements. In the present study we used the Brazilian Portuguese version described by Faria et al. (2015), maintaining the original timing protocol.

*Equipment*

A standard armchair with a seat height of approximately 46 cm and firm backrest was used as the starting position. A marker (adhesive tape or a small cone) was placed on the floor at a distance of 3 meters from the front edge of the chair. A digital stopwatch with 0.01 s precision was used for timing, and a clear, well-lit, non-slippery 4-meter pathway was required.

*Application protocol*

The participant was seated in the armchair with the back against the backrest, arms resting on the armrests, and feet flat on the floor. After standardized verbal instructions and one practice trial to ensure understanding of the task, the participant was asked to rise from the chair, walk at a comfortable and safe pace to the 3-meter mark, turn around the marker, return to the chair, and sit down again. The examiner used the verbal cue "Go" to start the trial. Walking aids were not permitted in order to obtain a measure of unassisted mobility. Two timed trials were performed, separated by at least 30 seconds of seated rest; the mean time of the two trials was used in the analysis.

*Scoring*

Timing started at the verbal cue "Go" and stopped when the participant was again fully seated with the back against the backrest. The result was expressed in seconds, with higher values indicating poorer mobility. Times above conventional cut-off values reported in the literature (e.g., >13.5 seconds in community-dwelling older adults) have been associated with increased risk of falls and were considered in the descriptive characterization of the cohort.

**4. Freezing of Gait Questionnaire (FOG-Q)**

*Overview*

The Freezing of Gait Questionnaire is a brief, clinician-administered, self-report instrument designed to characterize the presence, frequency, and severity of freezing of gait and related gait disturbances. The Brazilian Portuguese version validated by Baggio et al. (2012) was used in the present study.

*Application protocol*

The questionnaire was administered by a trained examiner in a quiet room, in the presence of the care partner whenever possible to support recall and minimize information bias. The examiner read each of the six items aloud and clarified terminology when needed, without suggesting any specific response. Items addressed: (1) general gait difficulty, (2) gait difficulty affecting daily activities, (3) sensation of feet getting glued to the floor while walking, turning, or trying to initiate walking, (4) duration of the longest freezing episode, (5) presence of start hesitation, and (6) presence of turning hesitation.

*Scoring*

Each item was scored on a 5-point ordinal scale ranging from 0 (no impairment) to 4 (most severe impairment), generating a total score from 0 to 24, with higher scores indicating more severe gait disturbance and freezing. A score of 1 or higher on item 3 was used as the operational threshold for the presence of freezing of gait, consistent with the conventions reported in the validation literature.

**5. Pull test (MDS-UPDRS item 3.12)**

*Overview*

Reactive postural control was assessed using the pull test as standardized in item 3.12 of the Movement Disorder Society-sponsored revision of the Unified Parkinson's Disease Rating Scale (MDS-UPDRS). The clinimetric properties of this maneuver in patients with parkinsonism and related disorders are detailed in Bloem et al. (1998).

*Application protocol*

The participant was instructed to stand comfortably, with feet slightly apart and eyes open, on a flat, non-slippery surface in front of an unobstructed space of at least two meters. The examiner positioned themselves directly behind the participant and, in this study, applied the test without prior notice in order to simulate the type of unexpected perturbation that may precipitate falls in daily life and to avoid the learning effect that has been described when a familiarization trial is performed. A single, brisk, backward pull on the participant's shoulders was applied, of sufficient magnitude to elicit a postural reaction. The examiner remained ready to catch the participant at any moment to prevent an actual fall.

*Scoring*

Performance was rated on the 0-4 MDS-UPDRS scale: 0 (normal: recovers with one or two steps), 1 (slight: 3 to 5 steps, recovers unaided), 2 (mild: more than 5 steps but recovers without assistance), 3 (moderate: stands safely but with absent postural response, would fall if not caught by the examiner), and 4 (severe: very unstable, tends to lose balance spontaneously or with only a very slight displacement). Scores of 2 or higher were interpreted as evidence of impaired postural reactions and were considered jointly with the BBS in the clinical characterization of the participants.

**References**

Van Loo MA, Moseley AM, Bosman JM, de Bie RA, Hassett L. Inter-rater reliability and concurrent validity of step length and step width measurement after traumatic brain injury. Disabil Rehabil 2003;25(21):1195-1200.

Miyamoto ST, Lombardi Junior I, Berg KO, Ramos LR, Natour J. Brazilian version of the Berg balance scale. Braz J Med Biol Res 2004;37(9):1411-1421.

Faria CDCM, Teixeira-Salmela LF, de Araujo PN, Polese JC, Nascimento LR, Nadeau S. TUG-ABS Portuguese-Brazil: a clinical instrument to assess mobility of hemiparetic subjects due to stroke. Rev Neurocienc 2015;23(3):357-367.

Baggio JAO, Curtarelli MB, Rodrigues GR, Tumas V. Validacao da versao brasileira da escala de congelamento da marcha. Arq Neuropsiquiatr 2012;70(8):599-603.

Bloem BR, Beckley DJ, van Hilten JJ, Roos RAC. Clinimetrics of postural instability in Parkinson's disease. J Neurol 1998;245(10):669-673.
